# A single-center experience of parathyroidectomy in 1500 cases for secondary hyperparathyroidism: a retrospective study

**DOI:** 10.1080/0886022X.2021.2016445

**Published:** 2022-01-30

**Authors:** Shasha Zhao, Wei Gan, Wenjia Xie, Jinlong Cao, Liang Zhang, Ping Wen, Junwei Yang, Mingxia Xiong

**Affiliations:** Department of Nephrology, Second Affiliated Hospital of Nanjing Medical University, Nanjing, China

**Keywords:** Parathyroidectomy, secondary hyperparathyroidism, complications, short-term outcome, hypocalcemia

## Abstract

**Background:**

Chronic kidney disease (CKD) is a global public health problem. With the deterioration of renal function, a certain proportion of CKD patients enter the uremic stage, and secondary hyperparathyroidism (SHPT) becomes a challenge. For refractory hyperparathyroidism, parathyroidectomy (PTX) plays a key role in reducing mortality and improving prognosis. Nevertheless, no consensus has been reached on the optimal surgical method. We aimed to provide evidence for the effectiveness of surgical treatment by summarizing the experience from our center.

**Methods:**

Clinical data from 1500 patients undergoing parathyroidectomy were recorded, which included 1419 patients in a total parathyroidectomy without autotransplantation (tPTX) group, 54 patients in a total parathyroidectomy plus autotransplantation (tPTX + AT) group, and 27 patients in the other group. Perioperative basic data, intact parathyroid hormone (i-PTH) levels, serum calcium levels, serum phosphorus levels, pathological reports, coexisting thyroid diseases, short-term outcomes and complications were analyzed. Moreover, postoperative complications were compared between the tPTX and tPTX + AT groups.

**Results:**

Parathyroid hormone, serum calcium and phosphorus levels decreased significantly post-surgery. Two patients died during the perioperative period. As the two most common complications, the incidences of severe hypocalcemia and hyperkalemia were 36.20% (543 cases) and 24.60% (369 cases), respectively. Pre-iPTH levels (OR = 1.001, 95% CI: 1.001–1.001, *p* < 0.01), serum alkaline phosphatase (ALP) levels (OR = 1.002, 95% CI: 1.001–1.002, *p* < 0.01) and the mass of excised parathyroid gland (OR = 3.06, 95% CI: 1.24–7.55, *p* = 0.02) were positively associated with postoperative severe hypocalcemia, while age and serum calcium were negatively associated with it. Pathological reports of resected parathyroid and thyroid glands indicated that 96.49% had parathyroid nodular hyperplasia, 13.45% had thyroid nodular hyperplasia, and 4.08% had thyroid papillary carcinoma.

**Conclusions:**

Parathyroidectomy is a safe and effective treatment for refractory secondary hyperparathyroidism. Severe hypocalcemia is the main complication, and coexistent thyroid diseases should never be neglected.

## Background

Secondary hyperparathyroidism, a common complication of chronic kidney disease, has gradually become important for the prolonged survival of patients with end-stage renal disease and has been accompanied by improvements in medical care [1]. There have been many subsequent studies on this disease since it was initially reported in 1934 [2]. To date, hyperphosphatemia, hypocalcemia, vitamin D deficiency, dysfunction of the fibroblast growth factor 23-klotho axis and other factors [3] are presumed to be associated with secondary hyperparathyroidism, which causes renal osteodystrophy, disorders of calcium and phosphorus metabolism, cardiovascular calcification, ectopic calcification, pruritus, anemia, myopathy, malnutrition and neuropathy [4]. In addition, it results in worse quality of life, increased cardiovascular and all-cause mortality [5]. Combined conservative therapy composed of appropriate diet, dialysis and medication is widely adopted. Nevertheless, diet control and adjustment of dialysis prescription contribute only slightly. Calcium-based phosphorus binders, active vitamin D and analogs may lead to hypercalcemia, which increases the risk of cardiovascular and ectopic calcification [6,7]; however, noncalcium-based phosphorus binders, vitamin D receptor agonists and calcimimetics have limited therapeutic effects on refractory secondary hyperparathyroidism, and they are costly.

Parathyroidectomy has gradually become an effective treatment for secondary hyperparathyroidism since it was first proposed by Stanbury in 1960 [8]. In the 2017 KDIGO Clinical Practice Guideline for CKD-MBD, parathyroidectomy was recommended to cure refractory secondary hyperparathyroidism [9]. Surgical options include total parathyroidectomy, total parathyroidectomy with autotransplantation and subtotal parathyroidectomy. However, none of the three has been proven to be optimal by randomized controlled trials. We performed 1500 parathyroid operations for patients suffering from refractory secondary hyperparathyroidism between September 2008 and October 2019. On this basis, our study was designed to provide evidence for surgical treatment by summarizing the experience from the past 1500 surgical cases of our center, recording pre- and post-operative complications, and estimating short-term outcomes.

## Methods

### Participants

This was a retrospective analysis of 1500 patients undergoing parathyroidectomy between September 2008 and October 2019 for secondary hyperparathyroidism. The indications for surgery were as follows: (1) serum intact PTH level > 500 pg/ml on two or more occasions after regular drug treatment; (2) parathyroid nodular or diffuse hyperplasia identified by ultrasound and sestamibi scan; (3) patients with related symptoms such as bone and joint pain, pathologic fractures, severe pruritus, and restless legs syndrome [10].

All procedures performed in the study were conducted in accordance with the ethical standards of the institutional and/or national research committee and with the Declaration of Helsinki. Our research was approved by the ethics committee of the Second Affiliated Hospital of Nanjing Medical University (ethical number: 2018KY110). There was no commercial sponsorship.

### Operative approach

Of 1500 patients, the numbers of tPTX, tPTX + AT and sPTX patients were 1419, 54 and 7, respectively. Thirteen patients underwent parathyroidectomy of residual parathyroid in the neck. Six patients underwent parathyroidectomy of forearm transplanted parathyroid tissue, and only one underwent total parathyroidectomy and parasternal auto-transplantation. None of Intraoperative Parathyroid Monitoring, Intraoperative Neuromonitoring or frozen section were used for identifying parathyroid glands or preservation of recurrent laryngeal nerves during the operation. We regularly placed cervical drains at both edges of the wound.

### Data collection

Collected data comprised patients’ gender, age, dialysis history, pre-and post-operative blood measurements, complications, corresponding therapies, and outcomes. Blood measurements included levels of preoperative hemoglobin, serum albumin, serum alkaline phosphatase, iPTH, serum calcium, and serum phosphorus. Serum calcium and phosphorus levels were also measured 1 h, 1 day, 2 days and 3 days after the operation. Postoperative severe hypocalcemia was defined as serum total calcium below 1.875 mmol/l 72 h after the operation [11]. For patients with serum albumin below 40 g/l, serum calcium was corrected using the following formula: Corrected calcium (mmol/l) = Total calcium + 0.08 × [40 − Serum albumin]. Postoperative hyperkalemia and severe hyperkalemia were defined as serum potassium above 5.5 mmol/l and 6.5 mmol/l, respectively, after surgery.

### Perioperative management

A central venous catheter was routinely inserted for intravenous calcium supplementation on the day before the operation. Intravenous calcium supplementation started immediately after surgery with 10% calcium gluconate and normal saline restricted to a ratio of 1:1 and a speed of 50–100 ml/h. Calcium carbonate in conjunction with active vitamin D was added after recovery of gastrointestinal function. The proportion of intravenous calcium supplementation was gradually reduced if serum calcium stabilized above 2.2 mmo/l. Heparin-free or regional citrate anticoagulation dialysis was performed 72 h after the operation.

### Statistical analysis

Statistical analysis was performed with SPSS 24 for Windows. Data are reported as the mean (SD) for continuous variables with a normal distribution and were compared by using paired *t*-tests. Continuous variable data with a skewed distribution are presented as the median (interquartile range) and were compared by using a nonparametric test. The chi-square test was used for the comparison of categorical variables. A binary logistic regression model was used to analyze the risk factors for severe hypocalcemia and hyperkalemia after tPTX. All independent variables were screened for collinearity, and variables with a *p*-value of less than 0.1 in the single‐factor analysis were chosen for further binary logistic regression. Forward stepwise regression was used to identify risk factors for severe hypocalcemia. Statistical significance was defined as *p* less than 0.05.

## Results

### Characteristics of the study population

The study population comprised 1500 patients (851 men and 649 women) with a mean age of 47.12 ± 10.90 years who were on long-term dialysis (93.43.0 ± 43.77 months). The biochemical profiles of our patients are presented in Table 1.

**Table 1.** Patient characteristics and baseline data.

Characteristics	Range or *n* (%)
Female gender	649 (43.27%)
Age (years)	47.12 ± 10.90
Dialysis vintage (months)	93.43 ± 43.77
Preoperative-iPTH (pg/ml)	1412.40 (961.80–2118.00)
Serum calcium (mmol/l)	2.46 ± 0.24
Serum phosphorus (mmol/l)	2.23 ± 0.55
Alkaline phosphatase (u/l)	202.50 (116.53–459.53)
Serum albumin (g/l)	41.98 ± 4.99
Hemoglobin (g/l)	107.48 ± 19.80

Continuous data are presented as the mean ± SD when normally distributed and as median (interquartile range) when skewed; iPTH, intact parathyroid hormone.

### Short-term outcome

All participants experienced a significant decrease in iPTH, serum calcium and serum phosphate. The median iPTH at 24 h after surgery was reduced to 2.90 pg/ml; additionally, their serum calcium dropped to an average of 2.09 mmol/l, and their serum phosphorus showed a declining trend 3 days after surgery, as shown in Table 2.

**Table 2.** Pre- and post-operative iPTH, serum calcium and phosphorus.

Variable	Preoperative	Postoperative	*p*-Value
iPTH [pg/ml, *M* (1/4, 3/4)]	1412.40 (961.80, 2118.00)	1 h 50.50 (31.70, 78.50)	<0.01
		1 d 2.90 (1.23, 7.98)	<0.01
Serum calcium (mmol/l)	2.46 ± 0.24	1 h 2.35 ± 0.25	<0.01
		1 d 2.09 ± 0.34	<0.01
		2 d 2.23 ± 0.34	<0.01
		3 d 2.21 ± 0.30	<0.01
Serum phosphorus (mmol/l)	2.24 ± 0.56	1 h 2.07 ± 0.57	<0.01
		1 d 1.77 ± 0.56	<0.01
		2 d 1.41 ± 0.46	<0.01

iPTH, intact parathyroid hormone; M, Median; h, hour; d, day; Normal ranges; iPTH, intact parathyroid hormone; iPTH (12–88 pg/ml); ALP (35–100 U/l); hemoglobin (120–160 g/l for male and 110–150 for female); albumin (40–55 g/l); phosphorus (0.85–1.51 mmol/l); and calcium (2.15–2.55 mmol/L).

### Complications and therapies

There were two deaths during the perioperative period, and the causes were acute cardiac tamponade and septic shock (biliary tract infection). Of 49 postoperative cervical hematoma cases, 34 cases were managed by local compression hemostasis, and 15 cases underwent debridement and suturing for massive hemorrhage. No case of suffocation or hemorrhagic shock was observed. 62 patients suffered from postoperative infections as follows: respiratory tract infection (52 patients), digestive tract infections (1 patient), surgical site infection (5 patients), femoral vein catheter-related infections (3 patients), and infection of cervical drainage tubes (1 patient). All infected cases healed after antibiotic therapy and other treatments except for one that died of septic shock (as mentioned above); 37 cases with recurrent laryngeal nerve injury characterized by hoarseness without dysphagia or dyspnea recovered after treatment with trophic nerve medicine. Sixteen cases of new-onset arrhythmia were found after surgery: atrial fibrillation (12 cases); ventricular premature beats (2 cases); supraventricular tachycardia (2 cases). All patients with new-onset arrythmia were converted to sinus rhythm after treatment. New-onset heart failure developed in 4 cases and was resolved by dialysis. 12 patients underwent arteriovenous fistula failure and subsequent operations for recreation, while 543 cases suffered from severe hypocalcemia. Numbness of limbs and lips were their main symptoms, which were managed by intravenous and oral calcium supplementation. No death caused by hypocalcemia-related laryngospasm occurred. No life-threatening malignant arrhythmia was observed in 369 cases with hyperkalemia, including 95 severe cases (as shown in Table 3).

**Table 3.** Complications in tPTX group.

Complication	Cases	Incidence (%)
Death	2	0.13
Hemorrhage	49	3.27
Infection	62	4.13
Severe hypocalcemia	543	36.20
Recurrent laryngeal nerve injury	37	2.47
Acute heart failure	3	0.20
Arrhythmia	16	1.07
Hyperkalemia	369	24.60
Severe hyperkalemia	95	6.33
Arteriovenous fistula failure	12	0.80

tPTX, total parathyroidectomy.

### Analysis of risk factors for hypocalcemia post parathyroidectomy

Considering the huge difference in quantity among different operative methods, only 1419 cases of total parathyroidectomy were chosen for analysis. Multivariate binary logistic analysis was used to identify risk factors for severe hypocalcemia. Variables with a *p*-value of less than 0.1 in single‐factor analysis, including age, preoperative iPTH levels, ALP levels, serum calcium levels, serum phosphorus levels, serum albumin levels, hemoglobin levels and the mass of excised glands, were chosen for further binary logistic regression, and forward stepwise regression was used to identify risk factors for severe hypocalcemia.

Research showed the following: Pre-iPTH (OR = 1.001, 95% CI: 1.001–1.001, *p* < 0.01), alkaline phosphatase (ALP) (CR = 1.002, 95% CI: 1.001–1.002, *p* < 0.01) and mass of excised parathyroid gland (OR = 3.06, 95% CI: 1.24–7.55, *p* = 0.02) were positively associated with severe hypocalcemia, while age (OR = 0.98, 95% CI: 0.96 ∼ 0.99, *p* < 0.01), serum calcium (OR = 0.02, 95% CI: 0.01 ∼ 0.05, *p* < 0.01) and hemoglobin (OR = 0.99, 95% CI: 0.98 ∼ 1.00, *p* = 0.02) were negatively associated with severe hypocalcemia, as shown in Table 4.

**Table 4.** Logistic regression analysis of severe hypocalcemia in the tPTX group.

Variable	OR	Single factor	*p*-Value	OR	Multiple factor	*p*-Value
		95% CI			95% CI	
Female gender (%)	1.16	0.93–1.4	0.20			
Age (years)	0.96	0.95–0.97	<0.01	0.981	0.967–0.994	0.006
Dialysis vintage (months)	1.00	1.00–1.00	0.25			
Serum calcium (mmol/l)	0.12	0.07–0.20	<0.01	0.038	0.018–0.80	<0.01
Serum phosphorus (mmol/l)	0.96	0.79–1.18	0.72			
Serum albumin (g/l)	0.92	0.90–0.95	<0.01			
Hemoglobin (g/l)	0.99	0.97–0.99	<0.01	0.99	0.98–1.00	0.02
Mass of excised gland (mg)	1.15	1.10–1.20	<0.01	3.06	1.24–7.55	0.02
Preoperative-iPTH	1.002	1.001–1.002	<0.01	1.001	1.001–1.001	<0.01
Alkaline phosphatase	1.004	1.003–0.004	<0.01	1.002	1.001–1.002	<0.01
Quantity of excised gland	1.23	0.95–1.60	0.12			

tPTX, total parathyroidectomy; OR, odds ratio; CI, confidence interval; iPTH, intact parathyroid hormone.

### Comparison of complications in the tPTX group and tPTX + at group

Demographic data, laboratory results and incidence of hypocalcemia and hyperkalemia were compared between the tPTX group and the tPTX + AT group. As shown in Table 5, there was a lower incidence of hypocalcemia in the tPTX group. Other indices showed no significant difference between the two groups.

**Table 5.** Comparison between tPTX and tPTX + AT groups.

	tPTX (1419 cases)	tPTX + AT (54 cases)	*p*-Value
Female gender (%)	609	24	0.88
Age (years)	47.14 ± 10.80	44.68 ± 11.78	0.11
Dialysis vintage (months)	92.56 ± 42.25	94.35 ± 53.85	0.77
Pre-iPTH [pg/ml, *M* (1/4, 3/4)]	1413.90 (967.53, 2119.13)	1313.50 (919.00, 2072.80)	0.93
iPTH (24 h after operation) [pg/ml, *M* (1/4, 3/4)], *M*(1/4, 3/4)]	2.7 (1.2–7.1)	6.2 (3.3–24.55)	<0.01
Serum calcium (mmol/l)	2.46 ± 0.23	2.54 ± 0.22	0.02
Serum phosphorus (mmol/l)	2.24 ± 0.55	2.19 ± 0.65	0.51
ALP [u/L, *M* (1/4, 3/4)]	199.00 (115.40, 454.00)	232.50 (125.00, 505.75)	0.93
Serum albumin(g/l)	41.96 ± 5.03	42.90 ± 4.22	0.19
Hemoglobin (g/l)	107.45 ± 19.64	105.78 ± 20.70	0.55
Severe postoperative hypocalcemia	496 (29.48%)	30 (46.94%)	<0.01
Severe postoperative hyperkalemia	88 (6.37%)	3 (6.12%)	0.63

tPTX, total parathyroidectomy; tPTX + AT, total parathyroidectomy with autotransplantation; Preoperative; iPTH, intact parathyroid hormone; ALP, alkaline phosphatase; M, median.

### Pathological reports of excised parathyroid glands

Pathological reports of 5361 excised parathyroid glands in 1500 operations indicated parathyroid adenoma (49), parathyroid nodular hyperplasia (5173), and parathyroid diffuse hyperplasia (75). In 1473 patients in the tPTX and tPTX + AT groups, 6 patients had 6 glands removed, 72 patients had 5 glands removed, 1294 patients had 4 glands removed, 76 patients had 3 glands removed. Nineteen patients had 2 glands removed, and one patient had 11 glands removed. For patients who had 2 or 3 glands removed, and further research identified residual parathyroid glands.

### Pathological reports of excised thyroid glands

In 1494 jugular parathyroidectomy operations, 201 cases were found to be thyroid nodular lesions, 64 cases were thyroid adenoma, 61 cases were papillary thyroid carcinoma (including 40 cases of papillary thyroid microcarcinoma). Lymph node metastasis existed in 13 cases.

## Discussion

Our study summarizes the experience of 1500 parathyroidectomy operations in a single-center, which comprised changes in postoperative iPTH, serum calcium, and serum phosphorus levels as well as perioperative complications, outcomes, pathological results of excised parathyroid glands and thyroid lesions, plus risk factors for severe hypocalcemia after surgery. The biggest highlight of this paper is not only the large sample size but also the large proportion of total parathyroidectomy in the selection of surgical methods. To the best of our knowledge, the number of total parathyroidectomy cases in our center is the largest. We hope the results of our research may offer a certain guiding significance for the selection of surgical modalities for parathyroidectomy. Parathyroidectomy is an effective method that can correct disorders of calcium and phosphorus metabolism, reduce the risk of fracture, relieve bone pain, alleviate anemia and decrease the risk of death [12–14]. This study described a drastic change in calcium and phosphorus metabolism. Serum calcium touched the bottom on the first day postoperation and then reached a stable state following high-dose calcium supplementation. There was a declining trend of serum iPTH and phosphorus. No large-sample research has confirmed the incidence and outcomes of long-term low serum iPTH and phosphorus.

As shown in our study, hypocalcemia was the most common postoperative complication, with a high incidence of 36.20%. Other observed complications included hyperkalemia (24.60%), hemorrhage (3.27%), infection (4.13%) and recurrent nerve injury (2.47%). To date, few large-sample studies have focused on complications of parathyroidectomy. Schneider et al. [15] analyzed the complications of 504 postoperative patients on tPTX + AT and reported the following: infection of incisional wounds (2%), hemorrhage (1%), acute cardiac decompensation (1.4%), and pneumonia (0.80%). In their research, the incidence of hemorrhage in 32 tPTX cases was 3.1%, which was slightly lower than ours. We routinely placed a central venous catheter and tPTX was our dominant operation type, which might increase the risk of infection and hemorrhage.

Postoperative severe hypocalcemia should be the focus of treatment and nursing because hypocalcemia could increase mortality and hospitalization [12,16]. In previous studies, male sex, younger age, higher preoperative iPTH, higher serum ALP and lower preoperative serum calcium were associated with an increased risk of postoperative severe hypocalcemia [17–19], and our study further reinforced this. In addition, the total mass of excised parathyroid glands instead of quantity was proven to be another risk factor. In addition, we identified a high preoperative hemoglobin level as a protective factor with minimal effect (OR = 0.99).

A higher incidence of hypocalcemia was seen in the tPTX + AT group, which might be explained by fewer patients and relatively conservative calcium supplementation. This reminded us to actively provide calcium supplementation even in the tPTX + AT group. In view of phlebitis and many other complications caused by peripheral venous calcium supplementation, we routinely placed a femoral vein catheter for intravenous calcium supplementation in conjunction with oral calcium supplementation and adjusted the dosage to maintain a stable serum calcium level. In this way, no patients died of severe hypocalcemia in the perioperative period.

Another issue that needs to be emphasized is postsurgical hyperkalemia, which has been reported in previous studies [20,21]. To date, several underlying mechanisms of postoperative hyperkalemia have been proposed. First, hyperkalemia is common among uremic patients, and tissue destruction caused by surgery further increases the risk. Another possible mechanism is as follows [20–24]: a sharp decline in serum iPTH levels leads to calcium influx into bone. Then, sodium ion influx into skeletal muscle cells increases through the membrane barrier action of the sodium–calcium exchanger. Increased sodium ion levels in skeletal muscle cells can reduce potassium ion influx by influencing the activation of the Na/K ATPase pump, which results in increased levels of extracellular potassium. To date, preoperative serum potassium, serum alkaline phosphatase, and dosage of calcium supplementation have been identified as factors influencing serum potassium levels after surgery [20,21,25]. To prevent cardiovascular events caused by hyperkalemia, a low potassium diet, increasing the dialysis frequency and routine electrolyte monitoring may play a certain role.

As reported in some studies, the probability of nodular hyperplasia of the parathyroid gland can be assessed by the duration of dialysis and the serum level of parathyroid hormone [26]. Pathological reports of 5361 excised parathyroid glands indicated parathyroid adenoma (49), parathyroid nodular hyperplasia (5173), and parathyroid diffuse hyperplasia (75). Reviewing the quantity of excised glands in each patient, 87.85% of cases had 4 glands removed, 6.45% of cases had less than 4 glands removed, and 5.36% of cases had 5 or more glands removed (including one patient who had 11 glands removed).

Coping with coexistent thyroid disease should be noteworthy. In addition to nodular hyperplasia and adenomatous hyperplasia, thyroid carcinoma was not uncommon, with a prevalence of 4.08%. Most identified thyroid carcinomas were papillary carcinomas, of which 65.67% were microcarcinomas, and lymph node metastasis existed in 21.31% of cases. This was partly explained by the fact that parathyroid papillary carcinoma was the most common parathyroid carcinoma, often asymptomatic and detected as incidental findings on autopsy or in surgical specimens [27]. Previous studies on thyroid carcinoma in patients with SHPT were mainly clinical analyses. Cristina et al. [28] collected clinical data from 217 patients undergoing parathyroidectomy for hyperparathyroidism (including 140 cases for primary hyperparathyroidism and 77 cases for secondary hyperparathyroidism), and the incidences of papillary thyroid carcinoma were 13.57% and 11.69%, respectively. There was no significant difference in incidence between the two groups. It seemed that patients with hyperparathyroidism were prone to thyroid cancer, but its pathogenesis has remained unclear until now.

Our study analyzed the curative effect of parathyroidectomy, common complications, outcomes, and pathological reports of resected parathyroid and thyroid tissues. However, there were many limitations to our study. First, the lack of long-term follow-up data is the biggest shortcoming of this study. We tried to collect sufficient data with a long-term follow-up but failed. It was truly a time- and labor-consuming project without a previously well-designed scheme. To overcome this defect, we are conducting a cohort study comparing long outcomes between the two operation methods. Other limitations still exist, such as the retrospective nature of the analysis from a single-center, incompleteness of clinical data, lack of hypocalcemia-related symptoms, and lack of improvement in bone pain and pruritus. Again, most of these data can be obtained by a well-designed prospective cohort study.

## Conclusion

In summary, although it may cause many complications, PTX remains a safe and effective therapy for SHPT under the premise of a strict selection of patients, careful perioperative management and proficient skill. Moreover, coexistent thyroid diseases can be found and treated concurrently.

## Ethical approval and consent to participate

This was a retrospective study using clinical data, and it did not involve further invasive intervention, treatment, or costs to patients. The study received a consent exemption and was approved by the ethics committee of Second Affiliated Hospital of Nanjing Medical University (ethical number: 2018KY110). All patients’ records were deidentified and analyzed anonymously. Our study was performed in accordance with the ethical standards of the institutional and/or national research committee and with the 1964 Helsinki declaration and its later amendments or comparable ethical standards. There was no commercial sponsorship.

## Data Availability

All data are available from the corresponding author by reasonable request.

## Abbreviations

CKD: Chronic kidney disease
SHPT: Secondary hyperparathyroidism
(ESRD): End-stage renal disease
PTX: parathyroidectomy
tPTX: total parathyroidectomy without autotransplantation
tPTX + AT: total parathyroidectomy with autotransplantation
i-PTH: intact-parathyroid hormone
ALP: alkaline phosphatase
